# Limb Lengthening after Primary Total Knee Arthroplasty: Customized Patient-Specific Instrumentation Does Not Affect Expected Limb Lengthening

**DOI:** 10.1155/2021/5573319

**Published:** 2021-02-22

**Authors:** Christopher Fang, Kenneth McAlpine, Michael Gustin, Ruijia Niu, David Freccero, Matthew Gordon, Eric L. Smith

**Affiliations:** ^1^Department of Orthopedic Surgery, New England Baptist Hospital, 125 Parker Hill Ave, Boston, MA 02120, USA; ^2^Department of Orthopedic Surgery, Boston Medical Center, One Boston Medical Center Pl, Boston, MA 02118, USA; ^3^Department of Orthopedic Surgery, Tufts Medical Center, 800 Washington St, Boston, MA 02111, USA

## Abstract

**Introduction:**

Expectations for limb length differences after TKA are important for patient perception and outcomes. Limb length discrepancies may occur due to postoperative leg length increases, which can lead to decreased patient functionality and satisfaction and even possible litigation. The purpose of this study is to examine the frequency and extent of limb lengthening among various preoperative deformities and between two different implant systems.

**Methods:**

Preoperative and postoperative full-length standing radiographs were obtained between August 2018 and August 2019 to measure mechanical axis and limb length of operative limbs. Demographic information such as age, sex, and BMI was also collected. Patients were grouped into categories for pre- and postoperative subgroup analysis: valgus, varus, customized implant, and conventional implant. Regression analysis was performed to evaluate significant relationships.

**Results:**

Of the 121 primary TKAs analyzed, 62% of the knees showed an increase in limb length after TKA, with an average lengthening of 5.32 mm. Preoperative varus alignment was associated with a mean lengthening of 3.14 mm, while preoperative valgus alignment was associated with a mean lengthening of 16.2 mm. Overall, there were no statistically significant differences in limb lengths pre- and postoperatively (*p* = 0.23) and no significant changes in limb length for any subgroup. Further, no variables were associated with limb length changes (*p* = 0.49), including the use of customized implants (*p* = 0.2).

**Conclusions:**

Limb lengthening after TKA is common and, on average, occurs more significantly in valgus knees. No significant difference in limb lengthening could be demonstrated using customized over conventional implants. Preoperative counseling is important to manage patient expectations.

## 1. Introduction

The effect of total hip arthroplasty (THA) on leg length changes has been extensively studied in the literature [1]. Despite reports of limb length changes following total knee arthroplasty (TKA) [2], minimal attention has been paid to changes in limb length that can be expected following TKA. The considerable effect of limb length discrepancy (LLD) on patient satisfaction, litigation, and outcomes following TKA warrants further study [3–5]. Given the high rate of success of TKA, patients undergoing the procedure expect excellent results and accurate expectations. However, limb length changes after TKA are not well characterized in the literature.

Changes in postoperative limb length have been shown to increase back pack, sciatica, gait disorders, and patient dissatisfaction [6]. This can occur after TKA due to limb lengthening of the operative leg [2, 6, 7]. Studies from 2012 and 2015 have shown that limb lengthening occurs frequently after TKA, though the extent of the lengthening is unclear [2, 8]. Additionally, recent advances in total joint technology may permit greater control over limb lengthening to allow for more precise outcomes. It has been proposed that patient-specific instrumentation (PSI) in TKA potentially permits a more accurate alignment, with comparable results for alignment restoration and component positioning [9, 10], augmented by three-dimensional computerized CT-scan planned custom implants for preoperative planning. This could potentially affect limb lengthening to a differing extent than conventional alignment techniques.

The purpose of this study is to examine limb lengthening and alignment after TKA in various conditions evaluating conventional and customized TKA implants. Our hypothesis is that overall, there will be an increase in limb length following TKA, and that customized TKAs will not have a different effect on limb lengthening than conventional implants.

## 2. Methods

### 2.1. Study Design

After Institutional Review Board approval was obtained, patients undergoing primary TKA were identified through a retrospective review of the database of a single fellowship-trained total joint surgeon from August 2018 through August 2019. Patients were included if they had pre- and postoperative full-length standing radiographs taken with an appropriate reference for image calibration. There were no cutoffs for patient age or BMI for this study. Exclusion criteria included lack of pre- or postoperative imaging, insufficient imaging (i.e., incomplete visualization of the entire limb), lack of magnification reference, flexion contractures >15 degrees, previous lower extremity fracture, or revision surgery. Due to ordering errors, lack of follow-up, or absence of a reference for image calibration, 5% of patients were excluded from the study. Films were sufficient for the remaining patients undergoing TKA.

All operations were performed by a single fellowship-trained surgeon using a medial parapatellar approach with standard flexion and extension gap balancing. Patients received either PFC Sigma (DePuy Orthopaedics, Inc., Warsaw, IN) cruciate retaining implants or customized, CT-based patient-matched implant with patient-specific instrumentation (PSI) (Conformis Inc., Burlington, MA). Indications for customized implants were patient preference, a functioning PCL (all CR), and anatomy that could exceed the limits of a conventional total knee system. Demographic information included age, sex, BMI, days from preoperative date to surgery date, days from postoperative date to surgery date, type of implant, and laterality.

Standing anteroposterior (AP) full-length digital images of the lower extremities were obtained before and after (6 months) the procedure according to institutional policies. Limb measurements were performed by a single researcher using TraumaCad online digital software suite (Brainlab AG, Germany) to determine the limb length and mechanical axis of the operative limb by taking the mean value of two measurements as the final data. Institutional protocol for the inclusion of a metal magnification ball (MB) for image calibration was followed in the majority of cases, and images were calibrated to this 25.3 mm ball. Cases in which MB imaging was absent were excluded.

Measurement tools available on TraumaCAD were used to improve the accuracy and consistency of measurements. A “Circle” tool was utilized to establish the center of the femoral head, and a “Line” tool was used to determine the center of the femoral intercondylar notch, tibial plateau, and plafond, with an “Angle” tool establishing the mechanical axis. Limb length was determined using the “Limb Length” function, which calculated total length using a point at the center of the femoral head to the tip of the medial tibial plafond.

All TKAs were grouped three times discretely for analysis: grouping one consisted of varus and valgus groups, grouping two consisted of “customized” and “conventional” groups, and grouping three consisted of “severe varus,” “severe valgus,” and “moderate malalignment” groups. Values greater than 10° in either varus or valgus qualified into “severe varus” or “severe valgus,” respectively, with the third group of “moderate malalignment” to capture the remaining knees in accordance with previously published studies [2, 8]. The mean limb lengthening and frequency of limb lengthening overall as well as the mean preoperative and postoperative alignment in each group were determined.

### 2.2. Statistical Analysis

Student's *t*-tests and Fisher's exact tests were appropriately used to compare demographic information between varus and valgus groups. Student's *t*-tests were performed to compare the difference in average limb lengthening within varus, valgus, customized, conventional, severe varus, severe valgus, moderate malalignment, and overall groups. Regression analysis was performed on leg length to control for variables including preoperative deformity, implant type, BMI, age, and sex. All statistical analyses were performed using Stata 12.0 software. Significance was defined as *p* < 0.05.

## 3. Results

We analyzed 121 primary TKAs (111 patients) during the study period. 101 TKAs were divided into the varus group, and 20 TKAs into the valgus group. Patient demographic features are presented in Table 1, which showed no significant differences between the groups (Table 1).

Overall, 62% of the 121 knees demonstrated increased limb length following TKA (Table 2). Average lengthening was 5.32 mm (SD: 4.42 mm; range: −6.2 to 51 mm) (Table 2). Mean preoperative alignment was 6.36° (SD, 0.82°), and postoperative alignment was 2.54° (SD, 0.33°) (Table 2). Mean change in alignment was 3.83° (SD, 0.74°) (Table 2).

60 (59.4%) of the 101 varus knees and 15 (75%) of the 20 valgus knees showed increased limb length after TKA (Table 2). Average lengthening of the varus group was 3.14 mm (SD: 4.92 mm; range: −6.2 to 17 mm), and it was 16.2 mm (SD: 9.79 mm; range: −4.1 to 51 mm) for the valgus group (Table 2). Average mechanical alignment for the varus group was 9.36° (SD, 0.59°) and 2.94° (SD, 0.34°) preoperatively and postoperatively, respectively, with a mean change in alignment of 6.42° (SD, 5.40°) (Table 2). Average mechanical alignment for the valgus group was 8.75° (SD, 1.5°) and 0.5° (SD, 0.79°) preoperatively and postoperatively, respectively, with a mean change in alignment of 9.25° (SD, 6.97°) (Table 2).

After discretely regrouping the 121 TKAs by type of implant, 66 (62.3%) of the 106 conventional knees and 9 (60%) of the 15 customized knees showed increased limb length after TKA (Table 2). The demographics between the two groups were similar with no significant differences. Average lengthening of the conventional group was 7.61 mm (SD, 4.81 mm), and it was 10.73 mm (SD, 10.22 mm) for the customized group (Table 2). Average mechanical alignment for the conventional group was 6.39° (SD, 0.93°) and 2.8° (SD, 0.35°) preoperatively and postoperatively, respectively, with a mean change in alignment of 3.58° (SD, 0.82°) (Table 2). Average mechanical alignment for the customized group was 6.2° (SD, 1.26°) and 0.67° (SD, 0.69°) preoperatively and postoperatively, respectively, with a mean change in alignment of 5.53° (SD, 1.19°) (Table 2). No significant changes in lengthening were found within each subgroup (Table 2).

The 121 TKAs were regrouped in a separate analysis, forming “severe varus,” “severe valgus,” and “moderate malalignment” groups. 38 (31.4%%) were found to have a severe varus deformity (Table 3). In the severe varus group, 71.1% of patients demonstrated increased limb length following TKA with an average lengthening of 13.9 mm (SD, 56.2 mm) (Table 3). Mean preoperative alignment was 15.34° (SD, 0.7°) compared to 4.68° (0.57°) postoperatively, with a mean change in alignment of 10.66° (SD, 0.78°) (Table 3).

Further, 43 (56.6%) were found to have a severe valgus deformity (Table 3). In the severe valgus group, 71.4% of patients demonstrated increased limb length following TKA with an average lengthening of 29 mm (SD, 44.4 mm) (Table 3). Mean preoperative alignment was 16.28° (SD, 1.71°) compared to 0.14° (SD, 1.22°) postoperatively, with a mean change in alignment of 16.43° (SD, 1.63°) (Table 3).

In the malalignment group, 76 (62.8%) demonstrated increased limb length following TKA with an average lengthening of 1.2 mm (SD, 43.5 mm) (Table 3). Mean postoperative alignment was 1.7° (SD, 3.1°) compared to 4.0° (SD, 5.1°) (Table 3). No significant changes in lengthening were found within each severe deformity subgroup (Table 3).

Regression analysis showed that the average limb lengthening in the valgus group was 11.57 mm (*p* = 0.337) greater than that in the varus group after adjusting for age, sex, BMI, and type of implant (Table 4). Limb lengthening had no relationship with age (*p* = 0.79), sex (*p* = 0.67), BMI (*p* = 0.73), or implant type (*p* = 0.2) (Table 4).

## 4. Discussion

In THA, limb lengthening effects can be minimized with preoperative planning in order to avoid potential complications [11]. In TKA however, the main operative goals of correcting the preoperative deformity and balancing ligaments may lower control and accuracy of leg length, often increasing operative limb length [2, 7]. Therefore, we sought to investigate the frequency and nature of limb length changes in TKA. We further established that an increase in limb length is routine after TKA. In agreement with past studies [2, 8], our overall values fell within the range of the established literature. Lang et al. and Tipton et al., in studies of 102 and 132 knees, found that 83.3% and 59.1% (compared to our 62%) demonstrated lengthening following TKA with an average increase of 6.3 mm and 4.38 mm (compared to our 5.32 mm) and mean postoperative alignment of 1.0° and 2.76° (compared to our 2.54°), respectively [2, 8].

Contradictory to Lang et al., none of our statistical tests were significant, showing no difference in pre- and postoperative limb changes for any subgroup [2], consistent with results found by Tipton et al. [8]. There were large mean magnitudes of leg length increase for the operative limb, but standard deviations contributed to nonstatistically significant results. The difference in operative leg length after surgery was not significantly different from the operative leg length before surgery. Though these authors [2] state that LLD is significant, it may not be clinically important if patients do not appreciate a difference in their limb length. Moreover, a study states that patients only perceive a leg length change of 2 cm or more [12], suggesting that the majority of patients in our study would not perceive a difference in their limb length after surgery. Our findings suggest that limb lengthening occurs frequently after TKA, but not to a statistically significant extent. As the temporal trend may indicate, there is potential that LLD may be decreasing over time as techniques and experience allow more control over leg length after surgery [2, 8]. As differences in leg length changes after TKA are reduced, it is likely that most patients will not perceive any leg length differences outside those with severe valgus knees.

The phenomenon of limb lengthening after valgus knees has been observed by several other authors [2, 8, 13]. The mechanism for these changes is unknown. Perhaps the lengthening could be due to less bone being resected during TKA in a valgus deformity knee. Further, the average change in alignment for valgus knees was also more profound, which could contribute to the overall increased length. This could potentially be avoided with CT-scan preoperative planning and awareness for valgus knee deformities [14]. Further research is needed on the exact mechanism and solutions to significant limb length changes for these patients.

Preoperative counseling is very important for managing expectations, especially in the stiff, severe valgus patient. It is logical that while patients might not perceive a leg length change of 2 cm or less, they might still feel small insignificant changes in their daily lives, despite the lack of functional deficits. While most patients are satisfied with the results of their operation relieving their debilitating pain and restoring function, there are still some patients that may be unhappy with their corrected, longer, and straighter leg. Therefore, it is of value to educate patients at increased risk for significant lengthening, such as those with a severe valgus deformity, to prevent unexpected outcomes.

Consistent with past studies, there was no association of any of the variables on leg length change [2, 8]. Our study furthers this evidence by showing that customized knees do not demonstrate significant leg length changes after surgery and have no relationship with limb lengthening. In other words, customized knees do not differ in postoperative LLD compared to conventional knee implants, further illustrating comparable outcomes for customized implants [10].

Chinnappa et al. found that perceived LLD was associated with decreased satisfaction and poorer functional scores but was not associated with radiographic LLD [13]. Hinarejos et al. found postoperative LLD as described by ≥10 mm to have significant worse functional outcome in KSS [15]. Kim et al. found that >15 mm LLD computer-assisted TKA showed lower functional outcome scores, although the authors state that the correlation was low [16]. Further, they found that results of the perception of LLD questionnaire for those with LLD >15 mm was significantly different from those with LLD <15 mm (23% vs 6%); however, results of the satisfaction questionnaires were similar between groups [16]. Regarding customized single use instruments, Romeo et al. found them to have economical and organizational benefits of reducing costs but noted a lack of difference from conventional instrumentation in terms of clinical outcomes such as radiographic parameters or knee scores [17]. Our study adds to the body of literature regarding LLD and expectations for patients after TKA, with customized implants showing no significant differences in limb lengthening over conventional implants.

The current study has several limitations, including its retrospective nature and relatively small sample size. The current study also relied on standing AP full-length radiography using commercially available software applied onto the radiographs to determine limb lengths and the degree of deformity in the coronal plane only. We did not evaluate changes in the sagittal plane, leaving any deformity that may have only been present in the coronal plane go unrecognized. Standing AP radiographs have been validated as accurate and reliable for use in determining LLD when compared to scanograms [18]. The measurement of leg length was shown to decrease as the degree of limb malalignment increased [18]. Flexion contracture preoperatively may affect limb length measurement, but a study has shown that the measurement is not affected by a contracture less than 15° [19]. Measurements were performed by a single researcher using an online digital software suite, potentiating influence by observer subjectivity. However, to reduce this influence, measurements were repeated a second time and the final data were the mean value of these numbers. The subset of the population that underwent primary TKA with PSI was lower than the other subsets, which could give a false sense of equal proportions across groups and increase the possibility of a type II error. Lastly, clinical significance of LLD after TKA is unclear [20]. Still, our study addresses an outcome variable that needs consideration and further research as subtle differences in pre- and postoperative limb lengths can be important for patient outcomes. Future research implementing prospective and meta-analysis studies are needed to fully understand the effect of subtle limb length differences.

## 5. Conclusions

In conclusion, our data further supports that limb lengthening is common after TKA. Though not statistically significant, it is reasonable that this increase in leg length for the operative limb represented a restoration of the normal joint space height and alignment of the native knee. This increase in leg length may not be clinically significant nor even perceived by the patient, which may allay negative outcomes seen with LLD. Customized PSI and implants do not affect LLD after TKA as compared to conventional implants.

## Figures and Tables

**Table 1:** Patient demographics.

Variable	Whole sample	Valgus (<0)	Varus (>0)	*p*
# pts	121	20	101	
Age (y)	66.1 (0.7)	66.1 (2.2)	66.1 (0.8)	0.99
BMI (kg/m^2^)	33.2 (0.5)	32.5 (0.9)	33.3 (0.5)	0.49
Female	88 (72.7%)	16 (80.0%)	72 (71.3%)	0.42
Days preop	107.0 (9.6)	115.4 (25.8)	105.3 (10.4)	0.70
Days postop	46.9 (3.3)	47.9 (4.5)	46.7 (3.8)	0.89
Sigma	106 (87.6%)	19 (95%)	87 (86.1%)	0.27
Left	57 (47.1%)	12 (60%)	45 (44.6%)	0.21

**Table 2:** Measurement results.

Parameter	Varus (*n* = 101)	Valgus (*n* = 20)	Customized (*n* = 15)	Conventional (*n* = 106)	Overall (*N* = 121)
Preop alignment^*∗*^	9.36 (0.59)	8.75 (1.5)	6.2 (1.26)	6.39 (0.93)	6.36 (0.82)
Postop alignment^*∗*^	2.94 (0.34)	0.5 (0.79)	0.67 (0.69)	2.8 (0.35)	2.54 (0.33)
Change in alignment^*∗*^	6.42 (5.40)	9.25 (6.97)	5.53 (1.19)	3.58 (0.82)	3.83 (0.74)
Lengthening (mm)^*∗*^	3.14 (4.92)	16.2 (9.79)	10.73 (10.22)	7.61 (4.81)	5.32 (4.42)
*P* ^†^	0.52	0.11	0.31	0.12	0.23
Percentage lengthening	59.4%	75%	60%	62.3%	62.0%

^*∗*^Values are expressed as mean (SD). ^†^Comparison of preoperative and postoperative limb length within each subgroup. Alignments are expressed in degrees. Lengthening is expressed in mm.

**Table 3:** Measurement results stratified by degree of deformity.

Parameter	Moderate malalignment (*n* = 76)	Severe valgus (*n* = 7)	Severe varus (*n* = 38)
Preop alignment^*∗*^	4.0 (5.1)	16.28 (1.71)	15.34 (0.7)
Postop alignment^*∗*^	1.7 (3.1)	0.14 (1.22)	4.68 (0.57)
Change in alignment^*∗*^	2.3 (5.3)	16.43 (1.63)	10.66 (0.78)
Lengthening^*∗*^	1.2 (43.5)	29 (44.4)	13.9 (56.2)
*P* ^†^	0.80	0.13	0.14
Percentage lengthened	56.6%	71.4%	71.1%

^*∗*^Values are expressed as mean (SD). ^†^Comparison of preoperative and postoperative limb length within each subgroup. Alignments are expressed in degrees. Lengthening is expressed in mm.

**Table 4:** Regression analysis on leg length (R-square = 0.04, *p* = 0.49).

	Coefficient	95% confidence interval	*p* value
Preoperative deformity status			
Valgus	11.57	−12.19–35.31	0.337
Varus	Reference		

Implant type			
Sigma	17.89	−9.6–45.49	0.2
Customized	Reference		

Body mass index	−0.23	−1.53–1.07	0.73

Age	−0.09	−0.73–0.55	0.79

Sex			
Male	4.3	−15.36–23.96	0.67
Female	Reference		

## Data Availability

The authors can make data available on request through a data access committee. Contact Ruijia Niu at ruijia.niu@bmc.org to request the data.
